# Autofluorescence-Guided Surgery in the Management of Osteonecrosis of the Jaw: Correlation Between Bone Autofluorescence and Histopathological Findings in 56 Samples

**DOI:** 10.3390/life15050686

**Published:** 2025-04-23

**Authors:** Ilaria Giovannacci, Aurora Andrea Venuti, Luigi Corcione, Samir Nammour, Paolo Vescovi

**Affiliations:** 1 Oral Medicine and Oral Surgery Laser Unit, University Center of Dentistry, Department of Medicine and Surgery, University of Parma, 43125 Parma, Italy; ilaria.giovannacci@gmail.com (I.G.); paolo.vescovi@unipr.it (P.V.); 2 Department of Medicine and Surgery, Section of Human Pathology and Histopathology, University of Parma, 43125 Parma, Italy; lcorcione@ao.pr.it; 3 Department of Dental Science, Faculty of Medicine, University of Liege, 4000 Liege, Belgium; s.namour@uliege.be

**Keywords:** bone autofluorescence, VELscope, Er:YAG laser surgery, Nd:YAG laser photobiomodulation, MRONJ

## Abstract

(1) Background: Osteonecrosis of the jaw (ONJ) remains a challenging condition without a universally accepted treatment protocol. Surgical therapy, particularly Er:YAG laser-assisted surgery, has shown more predictable long-term results compared with non-surgical options. However, the identification of resection margins in ONJ surgery is complex and currently relies on the surgeon’s intraoperative assessment, without standardization. Bone autofluorescence (AF) has been proposed as an intraoperative diagnostic tool for visualizing necrotic bone; under VELscope (LED Medical Diagnostics Inc., Barnaby, BC, Canada) illumination, healthy bone exhibits hyperfluorescence, while pathological bone appears dark brown/black (loss of autofluorescence, LAF). (2) Methods: 22 patients with ONJ requiring surgical therapy were included. After bone exposure, VELscope system was used to induce and visualize bone AF. Areas exhibiting absent or pale AF were identified as necrotic and removed; additional samples were collected from adjacent hyperfluorescent regions. (3) Results: Histopathologic evaluation of 56 specimens were conducted; 35 hypofluorescent samples were found to be necrotic bone tissue; in the 21 hyperfluorescent samples, 86% demonstrated normal, vital bone. The correlation between fluorescence and bone vitality was highly significant (*p* < 0.0000001). (4) Conclusions: Our data show that AF-guided surgical resection, combined with Er:YAG laser-assisted surgery, may improve clinical outcomes.

## 1. Introduction

Osteonecrosis, also known as avascular necrosis, is a broad term encompassing various conditions where bone and marrow cell death occurs due to ischemic processes [1,2].

Osteonecrosis of the jaw (ONJ) can be classified into several types based on its etiology: Medication-related osteonecrosis of the jaw (MRONJ), osteoradionecrosis (ORN), traumatic osteonecrosis, non-traumatic osteonecrosis, and spontaneous osteonecrosis [3].

MRONJ is a severe bone disease that can affect the jawbones of patients receiving antiresorptive drugs, such as bisphosphonates and denosumab, and/or other drugs defined as “biologic” or “target” anti-neoplastic drugs (particularly those with anti-angiogenic activity) such as bevacizumab, sunitinib, sorafenib, and aflibercept [4].

If a history of radiotherapy to the head and neck region exists, the diagnosis should be osteoradionecrosis [5,6]. Radiation causes inflammation and occlusion of the blood vessels that supply the bone, leading to avascular necrosis characterized by hypoxic, hypovascular, and hypocellular lesions [6].

The drugs and radiotherapy mentioned above are the primary risk factors for the onset of ONJ. However, the literature also describes cases of ONJ arising from both traumatic (thermal, mechanical, or chemical damage) and non-traumatic causes. Among non-traumatic forms is osteopetrosis, also known as marble bone disease, a hereditary condition resulting from mutations that impair osteoclast function. This leads to excessive bone formation, which increases bone density and compromises blood supply [7]. The inadequate vascularity, particularly in regions like the jaw, predisposes these areas to a higher risk of osteonecrosis [8].

Osteonecrosis of the jaw represents a complex challenge in clinical practice and to date, its treatment is still debated as there is no consensus on a specific protocol to follow [9]. In the literature, various therapeutic approaches have been proposed, typically classified into non-surgical, surgical, or combined strategies [10,11,12,13,14,15,16]. Non-surgical methods include antiseptic mouth rinses, antibiotics, low-level laser therapy (LLLT), and other conservative treatments. Surgical options encompass local debridement, sequestrectomy, marginal resection, and segmental resection [10,17,18,19,20].

Surgical treatment, in combination with medical therapy, offers more predictable results than non-surgical therapy alone across all disease stages and in the long term [21,22,23,24,25].

In particular, Er:YAG laser-assisted surgery has significant advantages over conventional surgical techniques. These benefits may stem from specific mechanisms of the Er:YAG laser, such as tissue biomodulation, the creation of a surface more conducive to tissue adhesion, and its bactericidal action, which helps prevent secondary infections in patients with ONJ [26,27].

### Identification of Necrotic Bone Margins in ONJ

The major difficulty in surgical removal of ONJ is the accurate identification of necrotic bone margins. Currently, the differentiation between necrotic and viable bone relies primarily on pre-operative radiographic studies and the surgeon’s intraoperative assessment. However, these criteria are subjective and not reproducible.

Several authors have proposed autofluorescence (AF) guided surgery as a valid method to visualize necrotic bone during debridement or surgical resection [28,29,30,31].

AF is the property of biologic tissues to spontaneously emit light when illuminated by a light source. This property is related to the presence of endogenous molecules, called fluorophores. In bone tissue, AF is primarily due to type I collagen, while the mineral content of bone does not contribute to fluorescence [32,33,34].

Pathological processes can lead to changes in the concentration, distribution, and physic-chemical properties of endogenous fluorophores, consequently leading to changes in autofluorescence [35].

The Visually Enhanced Lesion Scope (VELscope system, LED medical Diagnostic Inc., Barnaby, BC, Canada), is a simple, non-invasive, handheld device that emits blue light in the 400–460 nm spectrum, thus enabling direct visualization of tissue fluorescence alterations.

Under VELscope illumination, healthy bone exhibits hyperfluorescence, while pathological bone loses its ability to emit fluorescence and appears dark brown/black (loss of autofluorescence, LAF) [29].

The purpose of this prospective study is to present a case series describing a new surgical approach for ONJ, utilizing autofluorescence to highlight surgical margins. Histopathological evaluation of both fluorescent and non-fluorescent bone was performed to establish a more accurate correlation between fluorescence and bone viability.

## 2. Materials and Methods

### 2.1. Study Design

This prospective, single-center study was approved by AVEN (Area Vasta Emilia Nord) Ethical Committee of University Hospital of Parma (Study number: 739/2022/TESS/AOUPR AFBONE).

The study included patients diagnosed with osteonecrosis of the jaw who required surgical treatment. Patients were excluded if the management of the ONJ lesion involved a nonsurgical approach. Comprehensive documentation was collected for each patient, including medical, pharmacological, and dental histories. Radiographic examinations, such as panoramic radiographs and computer tomography scans, were performed to evaluate the lesions.

A total of 22 patients were included in the present study. Of these, 16 (73%) were female and 6 (27%) were male, with a mean age of 72 ± 9.61.

Intraoperative assessment of autofluorescence was performed by a single experienced surgeon. The assessment was conducted under blinded conditions with respect to histopathological outcomes, although the surgeon was aware of the clinical diagnosis at the time of surgery.

### 2.2. Autofluorescence-Guided Surgery

All patients received combined antibiotic therapy with amoxicillin (2 g/day) and metronidazole (1 g/day), administered from 3 days before to 3 weeks after the surgery. No preoperative tetracycline labelling was performed.

Surgical procedures were performed under local anesthesia. After bone exposure through a mucoperiosteal flap, VELscope system (LED medical Diagnostic Inc., Barnaby, BC, Canada), was used to induce and visualize autofluorescence in bone tissue. Necrotic bone showed no or pale AF, making it easily distinguishable from viable tissue.

Osteotomy was performed using a Lindeman bur to delineate the boundaries of the bone block and ensure accurate removal of all necrotic tissue.

After the removal of the necrotic bone block, AF visualization was used to guide the osteoplasty of the marginal bone, performed with a traditional ball-shaped bur. This procedure is minimally traumatic to the soft tissues and effective in removing the sharp edges of the bone. According to the AF image obtained after osteoplasty, Er:YAG laser (Fidelis Plus, Fotona, Ljubljana, Slovenia; parameters: 300 mJ, 30 Hz, fluence of 60 J/cm^2^) was then utilized to vaporize the necrotic bone, continuing the procedure until a strong AF signal was detected.

Additional tissue samples were then collected from the adjacent areas that were hyperfluorescent under the illumination of the VELscope.

Histopathologic evaluation was performed for all samples to investigate whether correlation was present between bone vitality and fluorescence.

The intraoperative procedure was continued by performing irrigation with 10% povidone–iodine solution. Low-level laser therapy (LLLT; Neodymium: YAG [Nd:YAG] laser, 1064 nm; Fidelis Plus; Fotona; power, 1.25 W; frequency, 15 Hz; fiber diameter, 320 mm) was administered in nonfocused mode at 2 mm from the tissues for 1 min (power density 1562.5 W/cm^2^, fluence 7 J/cm^2^) and repeated 5 times.

Tension-free wound closure was achieved using a continuous locking suture.

The follow-up protocol included weekly visits during the first month, each accompanied by a photobiomodulation session. Subsequent follow-up visits were scheduled at two months, three months, six months, and one year post-surgery. At each visit, clinical improvement was assessed in terms of symptom resolution and mucosal healing.

**Figure 1:**
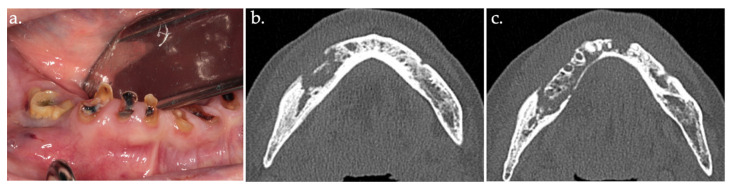
Intraoral view showing stage 2 MRONJ, with exposed sequestrum (**a**); CT scan image (**b**,**c**).

**Figure 2:**
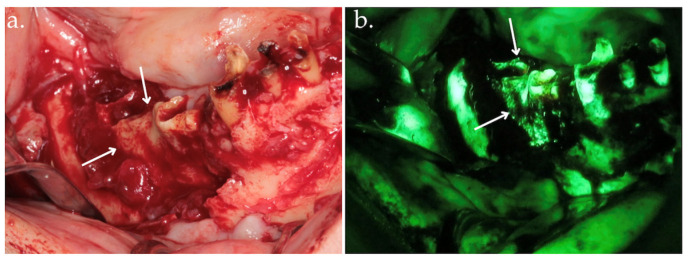
Clinical view after bone exposure through a mucoperiosteal flap with arrows indicating necrotic areas (**a**); direct intraoperative fluorescence examination showing hypofluorescent necrotic bone, highlighted by arrows (**b**).

**Figure 3:**
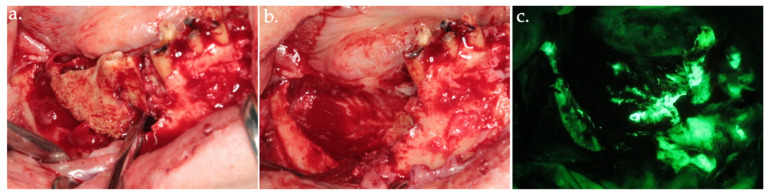
Delineation of margins and bone block removal in the clinical view (**a**,**b**), followed by autofluorescence imaging of the site post-excision (**c**).

**Figure 4:**
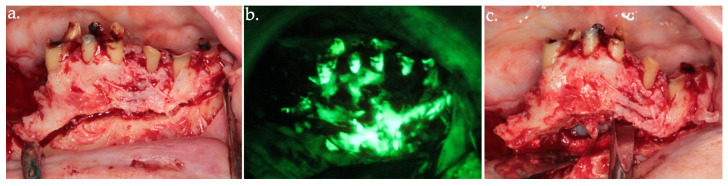
Osteotomy of the anterior necrotic bone segment in the clinical view (**a**) and its corresponding autofluorescence imaging (**b**). The complete removal of the bone segment is shown in (**c**).

**Figure 5:**
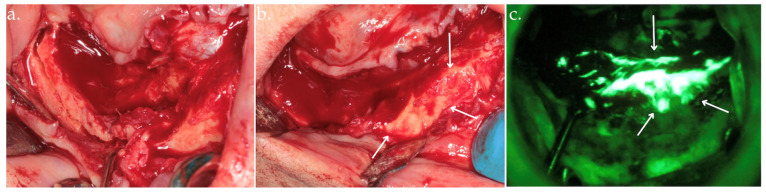
Osteotomy (**a**); osteoplasty using Er:YAG laser with arrows indicating viable bone (**b**), up to the identification of highly hyperfluorescent bone (arrows), confirming the clinical assessment (**c**).

**Figure 6:**
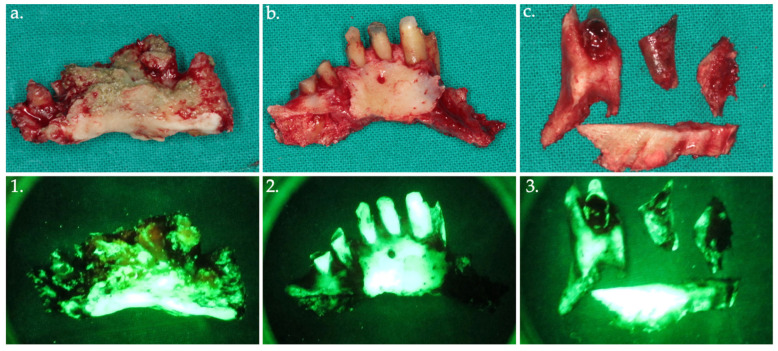
Bone fragments were removed and sent for histopathological analysis, shown in the clinical view (**a**–**c**) with their corresponding autofluorescence images (**1**–**3**).

**Figure 7:**
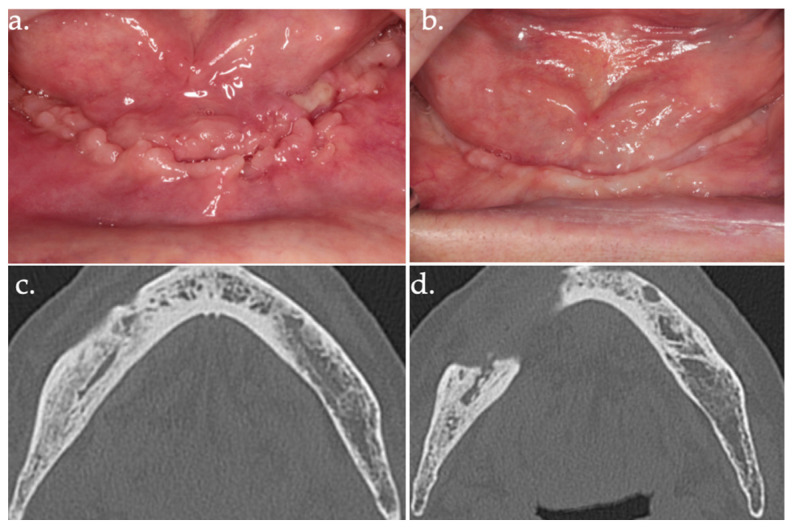
24 days follow-up (**a**); 17 months follow-up (**b**); follow-up CT scan at 18 months after surgery (**c**,**d**).

Since the clinical and surgical management was identical for all patients, the images presented (Figure 1, Figure 2, Figure 3, Figure 4, Figure 5, Figure 6 and Figure 7) illustrate the case of a representative patient.

### 2.3. Statistics

The results were expressed as percentages, median or mean ± SD, and/or range. Statistical analyses were performed using OpenEpi software (version 2.20), employing the uncorrected Chi-square test, Yates’ corrected Chi-square test, Mantel–Haenszel Chi-square test, Fisher’s exact test, and the mid-p exact test. The level of significance was set at *p* < 0.05.

## 3. Results

### 3.1. Patient Characteristics

Among the 22 patients, 9 (41%) had bone metastases due to malignancy, 4 (18%) had been diagnosed with multiple myeloma, 8 (36%) had osteoporosis, and 1 (5%) had osteopetrosis.

Regarding medication use, 13 patients (59%) were receiving bisphosphonates. One patient (5%) was receiving monoclonal antibodies, and six (27%) were treated with both bisphosphonates and monoclonal antibodies.

The patients’ demographic and clinical characteristics, along with potential risk factors for ONJ, are described in Table 1.

ONJ occurred in 63% (n = 14) of cases in the mandible and 36% (n = 8) in the maxilla. The distribution of ONJ stages among patients was as follows: 8 patients (36%) were diagnosed with stage I, 8 patients (36%) with stage II, and 6 patients (27%) with stage III.

### 3.2. Clinical Outcomes of Surgical Intervention

Surgical approach adopted induced complete healing in 21 sites (95%). The mean follow-up was 18.32 months (ranging from 2 to 36). Wound dehiscence requiring a second surgical intervention occurred in one (5%) patient; after the second surgery, the lesion achieved mucosal healing.

### 3.3. Histopathological Findings

**Figure 8:**
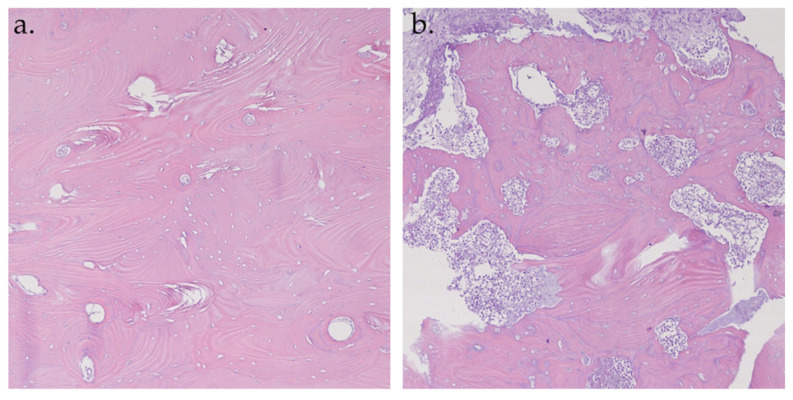
Histopathological analysis of hematoxylin–eosin specimens at 10× magnification. Hyperfluorescent sample: vital bone with vessels and osteons (**a**); Hypofluorescent sample: necrotic bone (**b**).

A total of 56 samples from 22 patients were analyzed. Of these, 35 samples were hypofluorescent. At the histopathological examination, all the hypofluorescent samples corresponded to necrotic bone tissue (Figure 8a). The remaining 21 samples were hyperfluorescent; 18 (86%) samples were normal structured vital bone (Figure 8b) and the other 3 samples (14%) were medullary bone tissue with foci of chronic osteomyelitis.

Histopathological findings and their correlation with autofluorescence analysis are shown in Table 2.

The correlation between fluorescence and bone vitality was highly significant, with the following statistical results: uncorrected Chi-square test *p* < 0.0000001, Yates’ corrected Chi-square test *p* = 0.000000222, Mantel–Haenszel Chi-square test *p* < 0.0000001, Fisher’s exact test *p* = 0.0001185, and the mid-p exact test *p* = 0.00005927.

## 4. Discussion

The surgical approach to managing osteonecrosis of the jaw is widely recognized for its high success rate across all stages of the disease [11,25,26]. Early-stage MRONJ surgery, in particular, yields more favorable outcomes as it prevents further disease progression and allows less invasive procedures that are less traumatic for the patient [36,37]. Various surgical techniques can be performed, ranging from superficial debridement to total resection of necrotic bone, with different success rates depending on the extent of necrosis and the surgical technique used.

Regardless of the approach, one principle remains consistent: the complete removal of necrotic bone is essential to prevent ONJ recurrence or progression. At the same time, it is vital to preserve as much healthy bone as possible. Overly aggressive removal of healthy tissue can weaken the jaw structure, potentially complicating future dental or prosthetic rehabilitation.

The identification of necrotic bone, especially along the margins of surgical resections, is highly complex and not coded. By definition, a resection margin should fall within the area of clinically and radiologically normal tissue immediately anterior, posterior, and/or underlying the pathologic bone tract to be removed, to ensure stable healing over time.

Several radiological tools, including computed tomography (CT), magnetic resonance imaging (MRI), fluoride positron emission tomography (PET), and orthopantomography, can, to some extent, help visualize the effective limits between viable and nonviable bone [38].

Surgical planning is typically guided by CT imaging, which is highly effective in delineating the extent of the disease [39,40]. MRI also represents a valuable diagnostic tool, offering superior sensitivity compared with CT in evaluating soft tissue changes surrounding the osteonecrotic area and in detecting bone marrow alterations during the early stages of osteonecrosis of the jaw [41]. However, both imaging modalities fail to identify the actual extent of the lesions. A study by Stockmann et al. demonstrated a statistically significant difference between lesion measurements obtained intraoperatively and those assessed with CT imaging [42]. As a result, intraoperative evaluation of the true extent of necrosis remains crucial. Surgeons rely on indicators of bone viability, including bleeding, bone structure, and color, to accurately assess the affected area [31,43].

All these criteria are subjective and make the procedure susceptible to arbitrariness [44]. This can lead to incomplete removal of necrotic tissue, which could promote disease progression or recurrence [45]. Furthermore, bone bleeding is not always correlated with histological findings of vital bone [28].

Thus, there is a clear need for advanced diagnostic tools that can identify surgical margins in real-time. Among these, fluorescence-guided surgery has proven to be a valuable tool for clearly visualizing resection margins and ensuring complete removal of necrotic bone, thus improving clinical outcomes [46].

The intraoperative application of autofluorescence has also been explored in other fields, such as intervertebral disc surgery. According to Hoell et al., the intraoperative use of AF allows precise differentiation between the disc material and the surrounding anatomical areas, as well as between the different fractions of the disc [47]. This approach provides valuable information about the quality and condition of the disc: healthy endplates are clearly identified by their intense fluorescence, while traumatized and degenerated discs appear significantly darker. Furthermore, the authors observed a statistically significant correlation between the number of viable osteocytes and the intensity of fluorescence, demonstrating a greater presence of viable osteocytes associated with a higher level of emitted photons.

Fluorescence-based identification of ONJ bone margins was initially achieved through tetracycline administration, representing the first use of fluorescence for evaluating resection boundaries [28,31]. Tetracyclines are a group of antibiotics that strongly chelate calcium and bind to the inorganic component of vital bone, but not to necrotic bone [48].

Preoperatively, patients received a tetracycline (e.g., doxycycline, minocycline) to allow the drug to incorporate into the bone matrix. During the operation, the surgical site was illuminated with a light source such as the VELscope.

When exposed to ultraviolet light with wavelengths between 365 and 490 nm, tetracycline emits a characteristic green fluorescence. Since the optimal excitation wavelength for visualizing tetracycline-bound bone is between 390 and 430 nm, its fluorescence can be detected using the VELscope, which emits blue light in the range of 400–460 nm [49].

However, as early as 1967, Prentice demonstrated that bone fluorescence primarily originates from collagen rather than from incidentally adsorbed substances [32,33]. His studies showed that the removal of minerals from bone sections or their exposure to weak acids, alkali metals, or organic solvents did not alter the autofluorescence signal. Type I collagen fluorescence is attributed to the presence of aromatic aminoacids such as phenylalanine, tryptophan, and tyrosine. Additionally, osteoid (non-calcified bone) exhibits a fluorescent signal, though this is significantly weaker than that of mature bone tissue.

In 2014, Pautke et al. reported that viable bone exhibited autofluorescence when illuminated with the VELscope [29]. This finding highlights the potential of the VELscope to assess bone viability without the need for tetracycline administration; healthy bone appears hyperfluorescent, whereas necrotic bone is hypofluorescent, pale, or non-fluorescent [50]. The loss of autofluorescence (LAF) observed in necrotic bone appears to result from the breakdown of collagen cross-links. The progressive degradation of collagen may account for the varying fluorescence intensities (hypo-, pale, or non-fluorescence) detected in necrotic bone [49].

Autofluorescence-guided bone surgery achieves success rates comparable to the tetracycline-based approach, with treatment success—defined as the absence of exposed bone after eight weeks—as the primary endpoint, and secondary outcomes including mucosal integrity, infection signs, pain, and loss of sensitivity [49].

A 2020 study by Ristow et al. [51] compared bone fluorescence in mini-pigs with MRONJ, to assess differences between autofluorescence and tetracycline-induced fluorescence in identifying viable and necrotic jawbone. The results showed no significant differences, confirming that both methods can identify healthy bone while areas lacking fluorescence correspond to necrotic tissue.

Microscopically, the current study highlighted a change in the arrangement and structure of collagen in the hypofluorescent necrotic areas, while in the hyperfluorescent viable bone, the collagen structure remained intact. Additionally, fluorescence was observed in cell-filled bone lacunae, which was absent in the necrotic areas (empty bone lacunae).

Avoiding tetracycline administration offers several advantages, including eliminating the need for preoperative drug administration and reducing reliance on patient compliance, without compromising fluorescence-based bone assessment, as autofluorescence is independent of drug bioavailability. Similarly, avoiding preoperative tetracycline administration reduces the risk of hypersensitivity reactions and adverse effects associated with antibiotic use.

The evaluation of autofluorescence can be compromised in the presence of hemorrhage due to the quenching effect of hemoglobin [52]. Hemoglobin exhibits an absorption band, known as the Soret band, between 400 and 440 nm, which absorbs a significant portion of the excitation light used for autofluorescence. As a result, the hemoglobin present in the tissue reduces the amount of excitation light available for inducing autofluorescence, leading to a diminished AF signal. Therefore, achieving optimal hemostasis prior to performing intraoperative autofluorescence assessment is crucial for ensuring accurate results.

In this study, autofluorescence was used at different stages as part of an innovative approach to the surgical management of ONJ. In the initial phase, it was used to accurately identify and demarcate resection margins, allowing a more precise and targeted surgical approach. Subsequently, it was useful during osteoplasty, guiding the surgeon in the removal of non-viable portions of bone. At the end of the osteoplasty procedure, autofluorescence was again used to detect any remaining hypofluorescent areas indicative of pathologic bone tissue. These areas can be removed minimally invasively via Er:YAG laser. Er:YAG laser (wavelength: 2.940 nm) is effective in treating hard tissues due to its high absorption of water and hydroxyapatite, the main components of bone tissue. The erbium laser causes “cold ablation”, interacting with tissue without inducing coagulation or carbonization. This increases its safety and reduces trauma compared with traditional instruments, thus accelerating the healing processes. Minimally invasive removal of necrotic areas, also known as Er:YAG laser bone vaporization, is a conservative surgery that involves making microperforations in the tissue (about 0.1 mm), thus facilitating the safe removal of all remaining necrotic tissue [26]. The surgery continues until an area of healthy, bleeding bone is exposed, promoting an environment that may facilitate future revascularization [27]. Furthermore, Er:YAG laser has bactericidal properties and biostimulatory effects, which promote faster healing of both soft and bone tissues, very useful in MRONJ surgery.

Management of MRONJ according to our protocol also involves a cycle of biostimulation via low-level laser therapy with Nd:YAG laser. LLLT represents an adjunctive treatment in medical or surgical management of MRONJ, at all stages of the disease, with proven benefits [53,54,55]. It induces biomodulation of soft and hard tissues with different effects, particularly anti-inflammatory, biostimulatory, antibacterial, and analgesic [56]. In bone tissue, LLLT causes an increase in the amount of mRNA which is utilized to synthesize type I collagen and thus stimulates bone tissue formation and repair [57,58].

It also promotes osteoblast differentiation, as shown by increased expression of osteocalcin (OCN), matrix metalloproteinase (MMP), and other markers of bone metabolism [59].

To investigate the correlation between fluorescence and bone viability, histopathological evaluation of fluorescent and nonfluorescent bone was performed. All hypofluorescent samples were found to be necrotic upon histopathological examination, exhibiting disruption of the bone architecture and collagen fiber destruction. The majority of hyperfluorescent samples (86%) were classified as vital based on histological analysis, displaying the typical structural features of viable bone tissue.

A small percentage of hyperfluorescent samples were identified as reactive medullary bone, which encircled the necrotic and inflammatory areas. These hyperfluorescent signals corresponded histologically to highly sclerotic medullary bone. The pronounced fluorescence is ascribable to the dense mineralization and compact structure typical of osteosclerotic bone, which occur as a consequence of altered bone remodeling dynamics. Histologically, osteosclerosis is defined by thickening of trabeculae, reduction of medullary spaces, and increased osteoblastic activity, often accompanied by aberrant mineral deposition [60]. These changes are not only structural but also biochemical, leading to the accumulation of autofluorescent molecules within the bone matrix, such as cross-linked collagen and advanced glycation end-products (AGEs) [61]. This biochemical profile contributes to the increased fluorescence signal observed during VELscope imaging.

In conditions such as chronic osteomyelitis, the interpretation of autofluorescence data must consider the presence of osteosclerosis to prevent potential misdiagnosis or overestimation of the severity of bone lesions. Therefore, the use of AF as a diagnostic tool should always be accompanied by a comprehensive assessment of the factors that may influence fluorescence, ensuring a more accurate interpretation of the underlying pathological changes.

One limitation of the present study was the absence of a control group consisting of patients who underwent conventional surgery without intraoperative autofluorescence guidance. It is methodologically challenging to evaluate surgical outcomes by isolating intraoperative autofluorescence as the sole variable of interest. In clinical practice, surgical success is influenced by a multitude of interrelated factors, including the extent and stage of osteonecrosis, anatomical variability, the surgeon’s technique, patient-specific healing capacity, and concurrent therapies (e.g., antibiotics, laser treatments, or photobiomodulation). As a result, attributing differences in clinical outcomes exclusively to the use of AF is not feasible within a complex surgical and therapeutic context. Compared with traditional surgery, the primary objective of intraoperative AF is not just to increase the effectiveness of necrotic bone removal but, more importantly, to enable a more conservative and tissue-preserving approach.

## 5. Conclusions

Autofluorescence has proven to be a reliable and reproducible method for accurately delineating surgical margins of necrotic bone, establishing its efficacy in clinical practice. Its minimally invasive nature improves surgical precision by reducing the risk of incomplete resection of necrotic tissue, thereby minimizing the risk of recurrence while avoiding unnecessarily extensive bone excision. This approach promotes preservation of healthy tissue and contributes to better clinical outcomes.

The integration of autofluorescence-guided surgery with laser technologies such as Er:YAG laser and biostimulation enables the development of an advanced therapeutic protocol, representing a significant advancement in the surgical management of ONJ. This multimodal approach facilitates the selective and highly precise removal of necrotic bone, optimizing postoperative healing processes and reducing morbidity. Furthermore, the combination of these minimally invasive techniques helps preserve healthy bone tissue, improving long-term clinical and functional outcomes.

Future proposals are to develop a system that measures AF in a numerical and objective way in real time during surgery.

## Figures and Tables

**Table 1:** Demographic data and potential risk factors.

Variable	Category	No. of Patients (%)
Age, years		72 ± 9.61
Gender	Male	6 (27%)
	Female	16 (73%)
Primary disease	Breast cancer	5 (23%)
	Multiple myeloma	4 (18%)
	Osteoporosis	8 (36%)
	Prostate cancer	3 (14%)
	Lung cancer	1 (0.05%)
	Osteopetrosis	1 (0.05%)
Medications	Zoledronate	7 (32%)
	Ibandronate	1 (0.05%)
	Neridronate	2 (0.05%)
	Denosumab (120 mg/month)	3 (0.05%)
	Alendronate	4 (18%)
	Bisphosphonates + monoclonal antibodies	6 (27%)
Comorbidities	Hypertension	8 (36%)
	Type 2 diabetes mellitus	2 (1%)
	Rheumatoid arthritis	1 (0.05%)
	Smoker	1 (0.05%)
	Ex-smoker (<10 years)	3 (14%)
	Long-term corticosteroid therapy	10 (45%)
ONJ local risk factors	Tooth extraction	6 (27%)
	Spontaneous	10 (45%)
	Perimplantitis	2 (1%)
	Denture pressure sores	4 (18%)

**Table 2:** Histopathological findings and their correlation with Autofluorescence analysis.

Histopathological Findings	Autofluorescence Analysis	
	Hypo-Fluorescent Bone	Hyper-Fluorescent Bone
Necrotic bone	35 (100%)	0 (0%)
Healthy bone	0	18 (86%)
Chronic osteomyelitis	0	3 (14%)

## Data Availability

Data are available on request from the authors.

## Abbreviations

The following abbreviations are used in this manuscript:

ONJ	Osteonecrosis of the jaw
MRONJ	Medication-related osteonecrosis of the jaw
ORN	Osteoradionecrosis
LLLT	Low-level laser therapy
AF	Autofluorescence
VELscope	Visually enhanced lesion scope
LAF	Loss of autofluorescence
CT	Computed tomography
MRI	Magnetic resonance imaging
